# Straggle in health care system for improving health status: what is vital in this challenge?

**DOI:** 10.1002/ehf2.12671

**Published:** 2020-04-19

**Authors:** Mostafa Mostafazadeh‐Bora, Mohsen Shahriari

**Affiliations:** ^1^ MSc of Medical‐Surgical Nursing, School of Nursing and Midwifery North Khorasan University of Medical Sciences Bojnurd Iran; ^2^ Nursing and Midwifery Care Research Center, School of Nursing and Midwifery Isfahan University of Medical Sciences Isfahan Iran

Li et al. published a noticeable article about a gap in heart failure management between Chinese societies in the latest issue of the *European Journal of Heart Failure*. They indicate a big gap in the care of patients with heart failure.^1^ Despite the progress in health care management, there are challenge in the prevention of disease severity. The role of health system in health care gap is vital and unknown.^2^

In worldwide, the high‐quality health service disturbance is essential for improving health status. This problem can assess two aspects including the provision of health supply and expertise by the health care system and the knowledge of the individual for utilizing it. 2 The various factor influence inequality in health, including health approach, sociocultural status, and health care system type. But the reasons of disparity in health were different among developing and developed countries.^3^

In developed countries, health care facilities for various people are public. On other hand, health structure in developed countries is proper and various socio‐cultural factors can influence on use of health services.^3^ For example, in the study of Osborn et al. about health care between 11 countries, the result of the study indicates that health care systems in developed countries does not our roles in access to inexpensive services with high quality correctly. In this study, individuals report several problems such as the lake of involvement patients in self‐management, inadequate management, and coordination for providing care especially in susceptible adults with poor income.^4^

Inequality in health care services in developing countries may be due to lack of effective health care system for providing health care supplies and health professionals. In fact, these countries need development resource and revision in the health care system. Also, health program in the developing countries is complex. These factors include governmental or financial unpredictability, lack of enough financial plans, and increasing individual without insurance that can defeat these programs.^5^

Finally, each health care system has a specific problem for solving health care status due to compound interaction between the national, regional, and political programs. All of the factors should support and amplify the access of the essential resource for improving knowledge and health status. Distribution of health services should have justice between population with various ethnicities and income level. Also, the national and regional program considers high‐risk people and supervises allocation of economic and health instrument for equality in health care.
